# Inflammation promotes oral squamous carcinoma immune evasion via induced programmed death ligand-1 surface expression

**DOI:** 10.3892/ol.2013.1238

**Published:** 2013-03-08

**Authors:** WANLU LU, LIBING LU, YUN FENG, JIAO CHEN, YAN LI, XIANGLI KONG, SIXIU CHEN, XIAOYU LI, QIANMING CHEN, PING ZHANG

**Affiliations:** State Key Laboratory of Oral Diseases, West China College of Stomatology, Sichuan University, Chengdu, Sichuan 610041, P.R. China

**Keywords:** inflammation, oral squamous carcinoma, tumor immune evasion, PD-L1, inflammatory cytokines

## Abstract

The association between inflammation and cancer provides a new target for tumor biotherapy. The inflammatory cells and molecules within the tumor microenvironment have decisive dual roles in antitumor immunity and immune evasion. In the present study, phytohemagglutinin (PHA) was used to stimulate peripheral blood mononuclear cells (PBMCs) to simulate the tumor inflammatory microenvironment. The effect of immune cells and inflammatory cytokines on the surface expression of programmed cell death-1 ligand 1 (PD-L1) and tumor immune evasion was investigated using flow cytometry (FCM) and an *in vivo* xenotransplantation model. Based on the data, PHA-activated, but not resting, immune cells were able to promote the surface expression of PD-L1 in Tca8113 oral squamous carcinoma cells via the secretion of inflammatory cytokines, but not by cell-cell contact. The majority of the inflammatory cytokines had no significant effect on the proliferation, cell cycle progression and apoptosis of the Tca8113 cells, although they each induced the expression of PD-L1 in a dose-dependent manner. In total, 99% of the Tca8113 cells expressed PD-L1 following treatment with the supernatant of PHA-stimulated PBMCs. The PHA-supernatant pretreated Tca8113 cells unusually induced Tca8113 antigen-specific CD8^+^ T cell apoptosis *in vitro* and the evasion of antigen-specific T cell attraction in a nude mouse tumor-bearing model. These results indicate a new mechanism for the promotion of tumor immune evasion by the tumor inflammatory microenvironment

## Introduction

In the 19th century, Rudolf Virchow observed that there were numerous leukocytes present within tumors, thus providing the first indication of an association between inflammation and cancer (1). More recently, inflammation has been shown to be a critical component of tumor progression (2). Furthermore, numerous cancer types have been observed to arise from sites of infection and inflammation (3). The development of cancer from inflammation may be a process driven by inflammatory cells, as well as a variety of chemical mediators (2–5). Inflammatory cells and cytokines establish a tumor inflammatory microenvironment, which is an essential component of all tumors and is involved in tumor progression by promoting proliferation, survival, immune evasion and migration (2,6,7). The immune cells and molecules secreted by these cells within the tumor microenvironment have decisive dual roles in anti-tumor immunity and immune evasion (8–10). Although this inflammatory response may suppress tumors, it may also facilitate cancer development and evasion via multiple signaling pathways (10,11). The association between inflammation and cancer has provided a new target for tumor biotherapy (12–14).

Programmed cell death-1 ligand 1 (PD-L1), also known as B7-H1, is a cell surface protein of the B7 family (15). Upregulation of PD-L1 in cancer cells has been observed in a variety of solid tumors, but not in normal tissue (16–19). The interaction between PD-L1 on cancer cells and programmed cell death-1 (PD-1) on immune cells has been shown to suppress activation and proliferation and induce apoptosis in the immune cells. Blocking the interaction of PD-L1 with PD-1 or the downregulation of PD-L1 surface expression in cancer cells promotes host antitumor immunity and inhibits the growth of tumor cells (20–23).

The mechanism by which PD-L1 expression is stimulated on the surface of tumor cells is not well understood. A previous study showed that interferon-γ (IFN-γ) is a potent stimulator of PD-L1 surface expression in various types of tumor cells in a time- and dose-dependent manner (24). In the present study, it was observed that the surface expression of PD-L1 on Tca8113 oral squamous carcinoma cells was increased by co-culture with activated immune cells, as well as by the major inflammatory cytokines that are secreted by immune cells following activation by antigens. These results provide evidence that inflammation promotes tumor evasion from the immune, suggesting that anti-inflammatory agents may offer new possibilities for cancer therapy.

## Material and methods

### Animals

Nude mice (body weight, 23–25 g) were purchased from the Animal Center of the Chinese Academy of Sciences (Shanghai, China) and housed in a specific pathogen-free (SPF) laminar flow room with a constant temperature (25–27°C) and humidity (40–50%). All experiments and animal care procedures were approved by the Animal Center of Sichuan University. The study was approved by the Ethics Committee of the State Key Laboratory of Oral Diseases, Sichuan University, Chengdu, Sichuan, China.

### Reagents

Phycoerythrin (PE)-labeled anti-human PD-L1, allophycocyanin (APC)-labeled anti-human CD8 and mouse IgG isotype control antibodies were obtained from eBioscience (San Diego, CA, USA). Recombinant human IFN-γ, interleukins (IL)-1, -2 and -6 and tumor necrosis factor-α (TNF-α) were purchased from R&D Systems (Minneapolis, MN, USA). The human tongue squamous carcinoma cell line, Tca8113, was obtained from the State Key Laboratory of Oral Diseases (Chengdu, China). The Annexin-V/FITC/Propidium Iodide (PI) Apoptosis Assay kit was purchased from Invitrogen (Carlsbad, CA, USA). Phytohemagglutinin (PHA) was purchased from Sigma-Aldrich (St. Louis, MO, USA).

### Cell culture

The Tca8113 cells were cultured in RPMI-1640 medium supplemented with 10% fetal bovine serum (FBS), 100 IU/ml penicillin, 100 *μ*g/ml streptomycin, 3% L-glutamine and 7.5% sodium bicarbonate (Gibco Life Technologies, Carlsbad, CA, USA). The cells were maintained as monolayers in 25-cm^2^ plastic tissue culture flasks at 37°C in a humidified atmosphere with 5% CO_2_. Exponentially growing cells were used in all experiments.

### Proliferation assay

The Tca8113 cells were seeded at a density of 4×10^4^ cells per well in 96-well plates. The cells were grown overnight and the medium was replaced with maintenance medium containing the desired concentrations of cytokines or medium. Cell viability was assessed subsequent to 72 h using the 3-(4,5-dimethyl-2-thiazolyl)-2,5-diphenyl-2H-tetrazolium bromide (MTT) colorimetric assay.

### Flow cytometry (FCM)

The pretreated Tca8113 cells were harvested and washed twice with FCM buffer (PBS with 5% FBS and 0.1% NaN_3_). Following incubation with PE-anti-human PD-L1 or isotype control antibodies for 30 min at 4°C, the cells were analyzed using a Beckman Coulter FC500 with Submit 5.2 software (Beckman Coulter, Miami, FL, USA).

### Cell cycle and apoptosis analysis

The analysis of the cell cycle distributions and the measurements of the percentage of apoptotic cells were performed by FCM. The Tca8113 cells were treated with the desired concentrations of cytokines or medium. Following treatment, the floating cells in the medium were combined with the attached cells collected by trypsinization. The cell cycle distribution and levels of apoptosis were analyzed using the Annexin V-FITC/PI Apoptosis kit (Invitrogen) and a Beckman Coulter FC500 within 1 h of staining. Cell cycle histograms were analyzed using Submit 5.2 and MultiCycle software. The percentages of apoptotic cells were analyzed using Submit 5.2 software.

### Preparation of activated T cells and supernatant

Briefly, peripheral blood mononuclear cells (PBMCs) from healthy human donors were isolated using Lymphoprep density gradient centrifugation. The PBMCs were plated at a density of 1×10^7^ cells per well in 6-well plates and stimulated with Tca8113 cell lysate or PHA. The tumor antigen-activated lymphoblasts were isolated by gradient centrifugation following stimulation with Tca8113 cell lysate for 7 days in culture medium (CM; RPMI-1640 medium containing 10% FBS, 100 IU/ml penicillin, 100 *μ*g/ml streptomycin and 1,000 IU rhIL-2). Following PHA stimulation for three days, the supernatants were collected and stored at −80°C as PHA-supernatant (PHA-sup).

### Analysis of the surface expression of PD-L1 on the Tca8113 cells

The Tca8113 cells (4×10^5^) were co-cultured with 1×10^6^ PBMCs per well in 6-well plates. The cells were grown overnight and the PHA, the PHA-supernatant, the desired concentrations of cytokines and 20 *μ*g/ml anti-cytokine antibodies were added to the corresponding cells. All cells were collected by trypsinization, stained with PE-anti-PD-L1 antibody and analyzed with FCM at day 3.

The Tca8113 cells were pre-labeled with carboxyfluorescein diacetate succinimidyl ester (CFSE; Invitrogen-Molecular Probes, Eugene, OR, USA). Briefly, the cells were washed and resuspended at a density of 5×10^6^ cells/ml in serum-free RPMI-1640, then incubated with a final concentration of 10 *μ*M CFSE at 37°C for 10 min with gentle agitation, washed twice with RPMI-1640 supplemented with 10% FBS and resuspended in RPMI-1640 supplemented with 10% FBS. The Tca8113 cells (4×10^5^) were co-cultured or separated by transwell inserts with 1×10^6^ PBMCs per well in 24-well plates, with or without 100 *μ*g/ml PHA, for 72 h. Following treatment, the attached cells were collected by trypsinization, stained with PE-anti-PD-L1 antibody and analyzed with FCM.

### CD8*^+^* T cell in vitro apoptosis assay

The activated T cells were then harvested and co-cultured with the Tca8113 cells that had been pretreated with inflammatory cytokines or PHA-supernatant for 18 h. Anti-PD-L1 blocking antibody (10 *μ*g/ml) was added into the indicated wells to block PD-1/PD-L1 interactions. All cells were then harvested and stained with APC-anti-CD8 antibodies and annexin V-FITC/PI. The apoptosis of the CD8^+^ T cells was calculated as the percentage of annexin V^+^/PI^+^ cells that were first gated on the CD8^+^ T cell population.

### In vivo immune evasion of Tca8113 cells pretreated with PHA

The Tca8113 cells were pretreated with PHA-supernatant for 72 h. Tumor cells (2×10^7^) were injected subcutaneously into nude mice to establish a subcutaneous xenotransplantation tumor model. Untreated control Tca8113 cells were injected around the neck and the PHA-supernatant pretreated Tca8113 cells were injected posteriorly. Tca8113 antigen-specific T cells (5×10^7^) or PBS as a control were injected via the tail vein at 0, 3 and 7 days subsequent to the tumor cells being injected. All the mice were sacrificed on the 21st day post-transplantation. Images of the tumors were captured, prior to the tumors being removed and weighed

### Statistical analysis

All values were expressed as the mean ± SEM. The data were analyzed by a one-way analysis of variance (ANOVA) followed by the Bonferroni test. P<0.05 was considered to indicate a statistically significant difference.

## Results

### PHA-activated immune cells promote expression of PD-L1 on Tca8113 tumor cells

**Figure 1:**
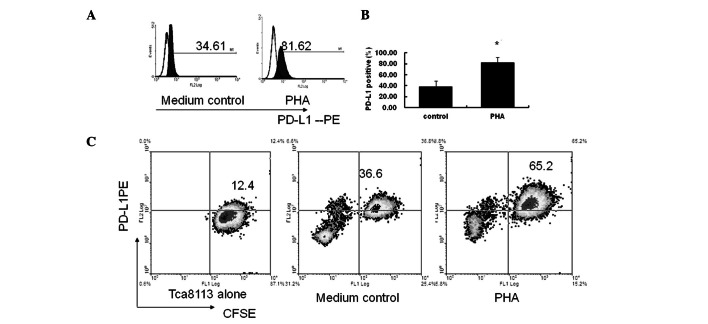
PHA-activated immune cells promote the expression of PD-L1 on Tca8113 cells. (A) PD-L1 surface expression in Tca8113 co-cultured with PBMC with or without PHA. The histograms indicate PD-L1 surface expression indicated by staining with isotype control antibodies and the grey histograms indicate staining with PE-anti-PD-L1 antibodies. The large numbers show the percentage of PD-L1-positive cells. (B) Error bars of (A) represent the mean ± SEM of three independent experiments. ^*^Significantly increased compared with that of cells from co-cultures without PHA treatment. (C) The Tca8113 cells were pre-labeled with CFSE and co-cultured with the PBMCs. The percentages of the CFSE^+^PD-L1^+^ Tca8113 cells were determined by FCM. Figures are representative of three independent experiments. PD-L1, programmed cell death-1 ligand 1; PHA, phytohemagglutinin; CFSE, carboxyfluorescein diacetate succinimidyl ester; PBMC, peripheral blood mononuclear cell; FCM, flow cytometry; PE, phycoerythrin.

PHA is a plant lectin, which acts as a mitogen to trigger the activation and cell division of T lymphocytes. The supernatant of the PHA-stimulated PBMCs contains a number of inflammatory cytokines, which are present in the inflammatory microenvironment in the body (25). In the present study, when the PBMCs were co-cultured with the Tca8113 cells and stimulated with PHA, the expression of PD-L1 by the Tca8113 cells was significantly increased compared with the cells cultured in medium alone (P= 0.006) (Fig. 1A and B).

In order to demonstrate that the observed PD-L1 was expressed primarily on the Tca8113 cells in the co-cultured system, the Tca8113 cells were pre-labeled with CFSE and co-cultured with PBMC again. Fig. 1 shows that there was no decrease in CFSE following 72 h in culture (Fig. 1C, Tca8113 alone). The percentage of the CFSE^+^PD-L1^+^ cells was significantly increased following stimulation with PHA compared with the control cells cultured in medium only (P= 0.006) (Fig. 1C, medium control and PHA). These results indicate that the PD-L1 was primarily expressed on the Tca8113 cells and not on the PBMCs. Only PHA-activated, but not resting, immune cells promoted the significant expression of PD-L1 on the Tca8113 cells.

### PHA-activated immune cells promote expression of PD-L1 on tumor cells via secreted inflammatory cytokines, but not by cell-cell contact

**Figure 2:**
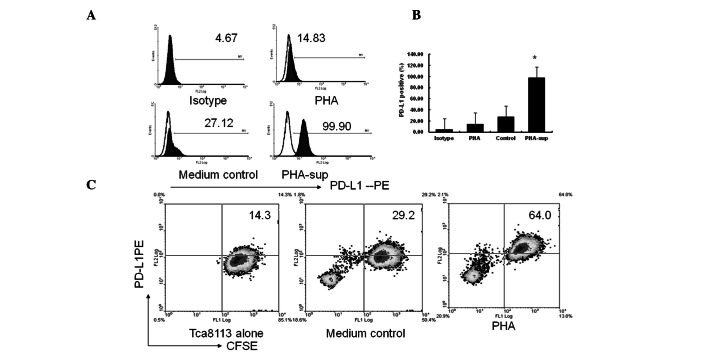
PHA-activated immune cells promote the expression of PD-L1 on Tca8113 tumor cells via the use of secreted inflammatory cytokines, not by cell-cell contact. (A) Histograms showing the surface expression of PD-L1 subsequent to the Tca8113 cells being exposed to 100 *μ*g/ml PHA or the supernatant of immune cells that were activated by PHA for 72 h. The open histograms indicate staining with isotype control and the solid histograms indicate staining with PE-anti-PD-L1 antibody. The numbers show the percentage of PD-L1-positive cells. (B) Error bars of (A) represent the mean ± SEM of three independent experiments. ^*^ indicates a value that was significantly increased compared with that of the cells from co-cultures without PHA treatment. (C) The Tca8113 cells were pre-labeled with CFSE and cultured with PBMCs separated by transwell inserts. The percentages of the CFSE^+^PD-L1^+^ Tca8113 cells were determined by FCM. Figures are representative of three independent experiments. PD-L1, programmed cell death-1 ligand 1; PHA, phytohemagglutinin; CFSE, carboxyfluorescein diacetate succinimidyl ester; PBMC, peripheral blood mononuclear cell; FCM, flow cytometry; PE, phycoerythrin.

PHA had no direct effect on the surface expression of PD-L1 when using the Tca8113 cells alone. However, the surface expression of PD-L1 on the Tca8113 cells was significantly increased following exposure to PHA-supernatant for 72 h (P= 0.001) (Fig. 2A and 2B). Similar to the PHA-supernatant, the CFSE^+^PD-L1^+^ cell (PD-L1^+^Tca8113) subset was significantly increased in the transwell culture system. However, no significant differences were observed when comparing the co-culture system with the transwell system (P=0.125) (Fig. 1C vs. Fig. 2C). These results indicate that activated immune cells significantly promote PD-L1 expression on Tca8113 cells via the use of secreted inflammatory cytokines, instead of cell-cell contact.

### IL-1α, IL-6, TNF-α and IFN-γ are the major inflammatory cytokines that promote PD-L1 expression on tumor cells

**Figure 3:**
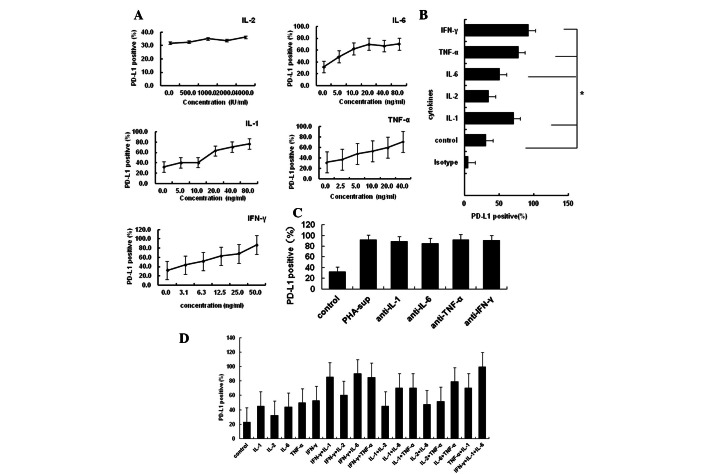
Inflammatory cytokines promote the surface expression of PD-L1 on Tca8113 cells in a dose-dependent and synergistic manner. (A) Surface expression of PD-L1 on the Tca8113 cells following treatment with increasing doses of inflammatory cytokines. (B) The Tca8113 cells were treated with 10 ng/ml IFN-γ, 10 ng/ml TNF-α, 10 ng/ml IL-6, 1000 IU/ml IL-2 or 20 ng/ml IL-1 for 48 h. Error bars represent the mean ± SEM of three independent experiments. (C) Surface expression of PD-L1 on the Tca8113 following treatment with 20% PHA-supernatant and blocking with 20 *μ*g/ml anti-cytokine antibody. (D) Surface expression of PD-L1 on the Tca8113 cells following treatment with the indicated inflammatory cytokines in combination. Data represent the mean ± SEM of three independent experiments. ^*^ indicates values significantly increased compared with untreated control. PD-L1, programmed cell death-1 ligand 1; PHA, phytohemagglutinin; IFN-γ, interferon-γ; TNF-α, tumor necrosis factor-α; IL, interleukin.

As IL-1α, IL-2, IL-6, TNF-α and IFN-γ are the major inflammatory cytokines secreted by immune cells activated by PHA, they are also located in the tumor microenvironment (25). Next, the present study examined whether these cytokines induced the expression of PD-L1 on tumor cells. The Tca8113 cells were exposed specifically to each of these cytokines at a desired concentration. The majority of these inflammatory cytokines induced PD-L1 expression on the Tca8113 cells in a dose-dependent manner, with the exception of IL-2 (Fig. 3A and B). However, 20 *μ*g/ml anti-cytokine antibodies against these cytokines did not block the expression of PD-L1 induced by PHA-supernatant (all P<0.05) (Fig. 3C). The majority of these cytokines had an additive effect with each other to promote the expression of PD-L1 (Fig. 3D). These results suggest that the inflammatory cytokine-induced surface expression of PD-L1 on Tca8113 cells is a result of multiple stimulatory factors.

### Effect of inflammatory cytokines on proliferation or apoptosis of tumor cells

**Figure 4:**
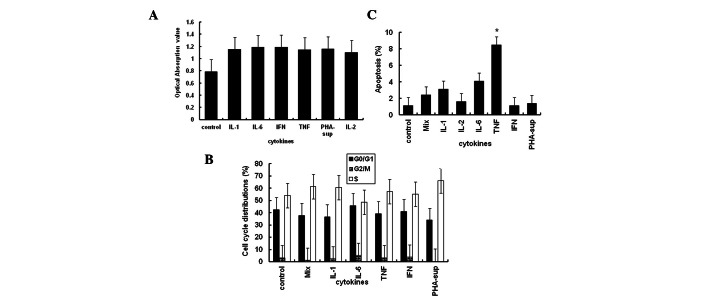
Effect of inflammatory cytokines on Tca8113 cell proliferation, apoptosis and cell cycle progression. (A) Cell viability was assessed using the MTT colorimetric assay at 72 h. (B) Tca8113 cells were collected by trypsinization and stained with PI. The percentages of cells undergoing apoptosis were analyzed on a Beckman Coulter FC500 with Submit 5.2 software. (C) The Tca8113 cells were collected by trypsinization and stained using an apoptosis assay kit. Cell cycle progression was analyzed on a Beckman Coulter FC500 with MultiCycle software. The results are representative of three independent experiments. ^*^ indicates values significantly increased compared with untreated control. PD-L1, programmed cell death-1 ligand 1; PHA, phytohemagglutinin; MTT, 3-(4,5-dimethyl-2-thiazolyl)-2,5-diphenyl-2H-tetrazolium bromide; PI, propidium iodide; IFN-γ, interferon-γ; TNF-α, tumor necrosis factor-α; IL, interleukin; sup, supernatant.

Previous studies have shown that the majority of inflammatory cytokines promote the proliferation of tumor cells (26). However, the results of the present study showed that 10 ng/ml of IL-1α, IL-2, IL-6, TNF-α and IFN-γ, the same concentration which was used to induce PD-L1 expression, had no significant effect on the proliferation of the Tca8113 cells (Fig. 4A) or the progression of the cell cycle (Fig. 4B). With the exception of TNF-α, all the cytokines investigated were also unable to induce apoptosis of the tumor cells (all P<0.05) (Fig. 4C).

### Inflammatory cytokines promote expression of PD-L1 on Tca8113 cells and induce the apoptosis of tumor antigen-specific T cells

**Figure 5:**
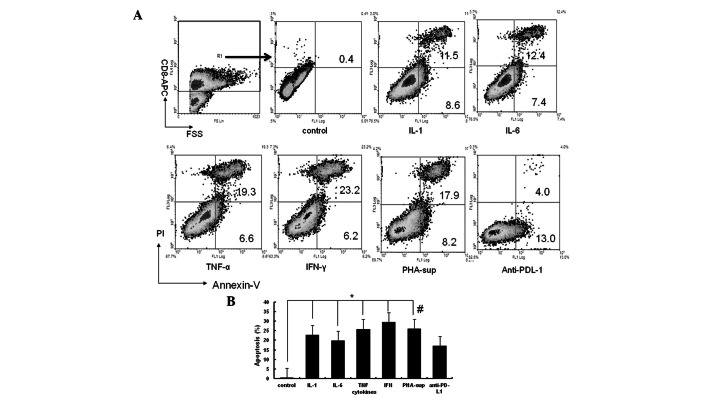
Inflammatory cytokines induce apoptosis of tumor antigen-specific T cells. Tca8113 antigen-activated human PBMCs were co-cultured at a ratio of 50:1 with the Tca8113 cells that had been pretreated with inflammatory cytokines. In the PHA-supernatant-pretreated sample, anti-PD-L1 mAb was added in an additional well for 16 h. The cells were then harvested and stained with anti-annexin V-FITC/PI and APC-anti-CD8 antibodies. (A) The apoptosis of the CD8^+^ T cells was analyzed on a Beckman Coulter FC500 with Submit 5.2 software gated first for the CD8-APC-positive group. The results are representative of three independent experiments. (B) Error bars represent the mean ± standard deviation of three independent experiments. ^*^P<0.05 vs. untreated Tca8113 cells, ^#^P<0.05 vs. anti-PD-L1 antibody block. PD-L1, programmed cell death-1 ligand 1; PHA, phytohemagglutinin; PBMC, peripheral blood mononuclear cell; IFN-γ, interferon-γ; TNF-α, tumor necrosis factor-α; IL, interleukin; PI, propidium iodide; sup, supernatant; FSC, forward angle light scatter.

As the PD-L1/PD-1 pathway is known to inhibit antitumor T cell-mediated immune responses, tumor antigen-specific T cells were co-cultured with the Tca8113 cells pretreated with inflammatory cytokines or PHA-supernatant. Anti-PD-L1 blocking antibody was added to demonstrate that the observed apoptosis was occurring via the PD-1/PD-L1 pathway. The percentage of CD8^+^ T cells undergoing apoptosis significantly increased subsequent to the co-culture with the Tca8113 cells pretreated with inflammatory cytokines (all P<0.05) (Fig. 5A and B). The apoptosis of the CD8^+^ T cells was significantly decreased following the addition of anti-PD-L1 to block the PD-1/PD-L1 pathway (all P<0.05) (Fig. 5B). These results suggest that inflammatory cytokines induce tumor cells to express PD-L1 to evade immune attraction.

### Tca8113 cells pretreated with inflammatory cytokines promote tumor immune evasion in vivo

**Figure 6:**
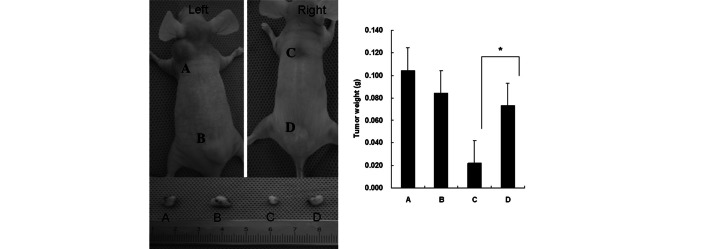
Tca8113 cells pretreated with cytokines promote tumor immune evasion *in vivo*. Nude mice were injected subcutaneously with 2×10^7^ PHA-supernata nt-pretreated (points B and D) or untreated control (points A and C) Tca8113 cells, followed by an intravenous injection of tumor antigen-specific T cells (right mouse) or PBS (left mouse) at three time points. All the mice were sacrificed at the 21st day post-transplantation and images were captured. The tumors were removed and weighed. The mice shown in the images are representative of each group (n=6). Error bars represent the mean ± standard deviation of the tumor weight. ^*^P<0.05 vs. the control Tca8113 group, which were treated with tumor antigen-specific T cells. PHA, phytohemagglutinin.

The untreated Tca8113 cells (Fig. 6A and C), or those pretreated with PHA-supernatant (Fig. 6B and D), were subcutaneously inoculated into nude mice. Certain mice also received Tca8113 antigen-specific T cells (Fig. 6, right mice). Pretreatment of the Tca8113 cells with PHA-supernatant did not affect the growth of the tumors *in vivo* (Fig. 6A vs. B). However, PHA-supernatant pretreatment of the Tca8113 cells (Fig. 6D) significantly promoted Tca8113 evasion from the Tca8113 antigen-specific T cell reaction.

## Discussion

The growth and reappearance of spontaneous tumors are considered to be the result of the resistance to therapy and evasion from the host immune reaction (27). A number of mechanisms for tumor immune evasion in the tumor microenvironment have been proposed (8,27,28).

Although immunotherapy has shown great promise in the treatment of human cancer, the resistance of cancer cells to this treatment remains a challenge. Clinical data have shown that PD-L1 is important for immune evasion by tumor cells. The expression of PD-L1 on tumors is markedly correlated with the survival of cancer patients. Targeting PD-L1/PD-1 interactions may improve the efficacy of adoptive cell therapies for chronic infections, as well as cancer (21,22).

However, the mechanism by which PD-L1 is expressed on tumor cells is not well understood. Studies have shown that IFN-γ is a potent stimulator of PD-L1 expression in various types of tumor cells (24). The association between inflammation and cancer was observed 150 years ago. It is now becoming clear that the tumor microenvironment, which is largely orchestrated by inflammatory cells and molecules secreted by immune cells, is essential for neoplastic proliferation, survival and migration (8,29–31). In the present study, the surface expression of PD-L1 on the Tca8113 cells was observed to be significantly increased following co-culture with PBMCs exposed to PHA (P=0.022), although PHA alone was unable to induce the expression of PD-L1. In addition, without the stimulation of PHA in the Tca8113/PBMC co-culture system, there was no significant increase in the expression of PD-L1. When the Tca8113 cells and PBMCs were separated in a transwell system, there was no significant change in the expression of PD-L1 on the Tca8113 cells compared with the increase observed when the Tca8113 cells were co-cultured with the PBMCs. The supernatant of the PBMCs exposed to PHA had the same effect on the surface expression of PD-L1 on the Tca8113 cells. These results demonstrate that activated immune cells, not resting immune cells, are able to promote the expression of PD-L1 via the use of secreted inflammatory cytokines, but not by cell-cell contact (Figs. 1 and 2).

PHA is a multiclonal T cell activator. When PBMCs are exposed to PHA, the CD4^+^ and CD8^+^ T cells are activated and secrete a number of inflammatory cytokines, including IL-2, IL-1α, IL-6, TNF-α and IFN-γ. The response of the T cells to PHA is a typical inflammatory response, similar to the reaction to a specific antigen (25). In the present study, the Tca8113 cells were treated with the supernatant of PHA-stimulated PBMCs, as well as recombinant human inflammatory cytokines, to simulate the inflammatory tumor microenvironment. The results indicated that PHA supernatant induced the positive expression of PD-1 on 99% of the Tca8113 cells (Fig. 2). With the exception of IL-2, all the inflammatory cytokines tested, including IL-1α, IL-6, TNF-α and IFN-γ, promoted the expression of PD-L1 on the Tca8113 cells in a dose-dependent and additive manner (Fig. 3A and D). However, anti-cytokine antibodies were unable to block the effect of the cytokines (Fig. 3C), suggesting that the cytokine-stimulated induction of the expression of PD-L1 on the Tca8113 cells is multifactorial.

It has been well-established that the inflammatory response in the tumor microenvironment is part of the normal host defense for the elimination of pathogens. As a result, the tumor microenvironment is composed of numerous innate and adaptive immune cells in addition to the cancer cells and their surrounding stroma. During the inflammatory response to the tumor, there is a balance between antitumor immunity and the promotion of tumor growth (32). In the tumor micro-environment, mature T cells exert tumor suppressive and tumor promoting effects that are determined by their effector functions. The inflammatory cytokines in the tumor microenvironment may be more relevant than the specific immune cell content. Various cytokines may either inhibit or promote tumor development and progression, regardless of their source (29).

The present study demonstrated that IL-1α, IL-2, IL-6, TNF-α, IFN-γ and the supernatant from the PHA-stimulated PBMCs had no effect on the proliferation of the Tca8113 tumor cells. With the exception of TNF-α, the cytokines also did not affect the apoptosis or cell cycle progression of the Tca8113 cells (Fig. 4). However, the Tca8113 cells pretreated with inflammatory cytokines significantly induced the apoptosis of the tumor antigen-specific CD8^+^ T cells *in vitro* and this effect was reversed by the anti-PD-L1 antibody (Fig. 5). Pretreatment of the Tca8113 cells with inflammatory cytokines did not affect their growth *in vivo* (Fig. 6A vs. B), but significantly decreased the antitumor effect of the tumor antigen-specific T cells (Fig. 6D). These results indicate a new mechanism for the promotion of tumor immune evasion by the tumor inflammatory microenvironment
